# Assessment and Evaluation of Oral Health in Orthodontic Patients: A Cross-Sectional Study

**DOI:** 10.7759/cureus.98194

**Published:** 2025-11-30

**Authors:** Himasagar Ellampalli, Veeresh Matmari, Shanthosh Raj Srinivasan, Jhansi Rani Lotavath, Periyasamy Sekar

**Affiliations:** 1 Dentistry, Employees State Insurance Corporation (ESIC) Medical College and Hospital, Chennai, IND

**Keywords:** fixed orthodontics, gingival bleeding index, malocclusion of teeth, oral hygiene status, orthodontic plaque index

## Abstract

Background: The management of good oral hygiene during fixed orthodontic treatment is crucial to avert the occurrence of problems like plaque formation and gingival inflammation. Fixed appliances promote plaque retention and thus predispose the patient to periodontal disease that may negatively impact the outcomes of the treatment.

Aim: This study aimed to evaluate changes in gingival bleeding, gingival inflammation, and plaque accumulation during the first month of fixed orthodontic treatment.

Materials and Methods: A total of 43 patients who received orthodontic fixed appliances at a tertiary healthcare facility in Chennai were selected. The patient's gingival health was measured three times, i.e., prior to the beginning of treatment (T0), during the setting of the appliance (T1), and one month into the treatment (T2). Gingival index (GI) and orthodontic plaque index (OPI) were used to assess the gingival health and the level of plaque. The analysis was done through IBM SPSS version 21.0 with a p-value set at 0.05, where the chi-square and Fisher's exact test were used.

Results: The GI scores before treatment and at the time of placement of appliances were stable, but showed a significant increase in inflammation within one month after initiation of treatment (p=0.004). The significant decline of gingival health in the first month compared to the baseline and the start of treatment was established by pairwise comparisons (p=0.017). The OPI scores showed a great increase in the amount of plaque accumulation after one month (p=0.039). These findings indicate the presence of plaque retention and early gingival inflammation in cases of fixed orthodontic appliances.

Conclusion: Fixed orthodontic treatment causes a considerable deterioration of gingival health and oral hygiene during the initial month of appliance placement. The prevention of periodontal complications and the optimization of orthodontic outcomes depend on early and continuous oral hygiene education, which reduces periodontal complications.

## Introduction

Good oral hygiene is a key part of successful orthodontic treatment. When patients begin fixed appliance therapy, the brackets, bands, and wires create new niches that easily trap plaque and make cleaning more difficult. This increase in plaque retention can set off a cascade of complications, ranging from gingival inflammation to enamel decalcification, caries, and halitosis [1,2]. These changes not only threaten oral health but can also reduce patient comfort and lengthen treatment time.

The shift in the oral environment following appliance placement is well documented. Plaque levels tend to rise, and studies have shown that the microbial balance of the mouth shifts toward more cariogenic and periodontopathogenic species [3,4]. Higher counts of *Streptococcus mutans* and *Lactobacillus* were reported throughout orthodontic treatment, which increases the risk of caries as well as periodontal breakdown [5]. These changes can promote the growth of periodontal pathogens such as *Porphyromonas gingivalis*,* Prevotella intermedia*,* Fusobacterium nucleatum*, and* Aggregatibacter actinomycetemcomitans,* which are strongly associated with gingival inflammation and early periodontal breakdown. Often, the very first clinical sign of poor plaque control is gingival inflammation, bleeding, and swelling that may appear within weeks of treatment initiation [6]. If ignored, these changes can progress into more serious periodontal problems and compromise the long-term stability of results [7].

Importantly, several researchers have drawn attention to the early phase of orthodontic treatment as being particularly critical. Lara-Carrillo et al. found that bacterial colonization and plaque accumulation rose significantly within just four weeks of appliance placement [8]. Likewise, Arab et al. [9] demonstrated that gingival indices increased within the first month, underscoring the need for preventive reinforcement early on. These findings suggest that clinicians should focus not only on long-term oral health during treatment but also on identifying the “high-risk window” shortly after appliances are fitted.

Despite the growing evidence, there is still relatively limited data on how quickly gingival bleeding, inflammation, and plaque accumulation change in the first month of fixed orthodontic therapy. Understanding these early responses is essential because they often set the stage for the patient’s periodontal health throughout treatment. With orthodontic treatment becoming increasingly common among both adolescents and adults worldwide, protecting periodontal health is not only necessary for treatment success but also for maintaining overall oral well-being [10]. 

The present study was therefore designed to assess and evaluate the gingival status during the first month of fixed orthodontic treatment. By focusing on this critical initial period, research gives insights into the early periodontal response and highlights opportunities for timely preventive interventions.

## Materials and methods

Study design and ethical approval

This study evaluated plaque accumulation and gingival health following the placement of fixed orthodontic appliances. Ethical clearance was obtained from the Institutional Ethical Committee (Approval No. IEC/2024/2/46). All participants were informed about the research process, and written consent was obtained prior to enrollment.

Study population and sample size

This cross-sectional observational study included 43 patients who reported to the Dental Outpatient Department of the Employees State Insurance Corporation (ESIC) Medical College and Hospital, a tertiary care hospital in Chennai, India, for fixed orthodontic treatment and were treated with stainless steel metal brackets of 0.022 slot. Patients were selected through convenience sampling.

The required sample size was calculated using G*Power software (version 3.1.9.7, Heinrich-Heine-University Düsseldorf, Germany) [11]. Based on previous studies, Yadav et al. [12] evaluated gingival index (GI) changes after orthodontic appliance placement; the pooled standard deviation and mean difference yielded an estimated effect size (Cohen’s d) of approximately 0.5. Considering an alpha error of 0.05 and a power of 90% (β = 0.1), the minimum required sample size was calculated to be 43 subjects.

Inclusion and exclusion criteria

Eligible participants were patients aged between 14 and 25 years who were scheduled to undergo fixed orthodontic treatment, were systemically healthy, and were willing to participate by providing informed consent. Patients were excluded if they were medically compromised or had systemic diseases affecting periodontal health, had received antibiotics or anti-inflammatory drugs within the past three months, were smokers or had deleterious oral habits, presented with periodontal disease at baseline, or declined to participate.

Clinical examination and study parameters

All participants received fixed orthodontic treatment using 0.022 × 0.028-inch MBT prescription pre-adjusted edgewise stainless steel brackets. The brackets were bonded using a light-cured adhesive (Orthofix, Lewisville, TX), following standard etching and bonding protocols. The initial aligning archwire used was 0.012-inch nickel-titanium (NiTi), selected to produce light and continuous forces appropriate for the initial alignment phase. All patients were treated by the same operator to eliminate inter-operator variability.

To minimize potential confounding factors, patients were selected based on similar baseline oral hygiene status and periodontal health. Variables such as type of malocclusion (crowding, spacing, or well-aligned arches), dietary habits, chewing patterns, and oral hygiene practices were assessed at baseline. All participants received standardized oral hygiene instructions, including brushing twice daily with fluoridated toothpaste and the use of interdental aids. They were instructed to maintain their regular diet and oral hygiene routine throughout the observation period.

To ensure data consistency, no professional cleaning or adjunctive periodontal therapy was performed during the one-month evaluation period.

Oral examinations were conducted using a mouth mirror and periodontal probe under adequate illumination. Two indices were used to assess gingival health and plaque levels:

GI was scored according to Löe and Silness criteria [6], recorded at three intervals: before treatment initiation (T0), at appliance placement (T1), and one month after treatment initiation (T2).

Orthodontic plaque index (OPI) was defined by Heintze et al. [13] and was used to measure plaque accumulation around orthodontic brackets, and was recorded at appliance placement (T1) and one month later (T2).

The GI and the OPI are widely used in orthodontic research. The GI does not require permission for use, while the OPI was described according to the original authors’ methodology without reproducing copyrighted figures or tables.

All examinations were performed by a single trained examiner. Intra-examiner calibration was conducted on 10 patients prior to the study, yielding a kappa value of 0.85, which indicated good reliability.

Statistical analysis

Data were compiled in Microsoft Excel (Microsoft Corp., Redmond, WA) and analyzed using IBM SPSS Statistics version 21.0 (IBM Corp., Armonk, NY). Descriptive statistics (frequency, percentage, mean, and standard deviation) were calculated. Differences in GI and OPI scores at different time intervals were analyzed using the chi-square test and Fisher’s exact test. A p-value of <0.05 was considered statistically significant.

## Results

All patients, including those with mild baseline gingival inflammation (GI score ≤2), were included to reflect the typical clinical population undergoing fixed orthodontic treatment (Table 1). No patients had moderate or severe gingivitis at baseline, ensuring that pre-existing inflammation was minimal. By assessing changes over time within the same patient (baseline vs. one month), the study evaluates the effect of appliance placement on gingival health rather than absolute disease prevalence. This within-subject comparison reduces selection bias and allows us to attribute observed increases in GI and OPI scores primarily to the introduction of fixed orthodontic appliances.

**Table 1:** Baseline table showing demographic characteristics SD: standard deviation; GI: gingival Index

Characteristic	Value
Total participants (n)	43
Age (years), mean ± SD	19.5 ± 3.2 (range 14–25)
Gender: male; n(%)	18 (41.9%)
Female	25 (58.1%)
Baseline GI	All participants had GI ≤ 2 (mild inflammation only)
Baseline gingivitis severity	No moderate or severe gingivitis observed
Oral hygiene status at baseline	Within normal/mild variation; no periodontal pockets detected
Inclusion of patients with mild baseline inflammation	Yes (to reflect typical orthodontic population)

**Figure 1:**
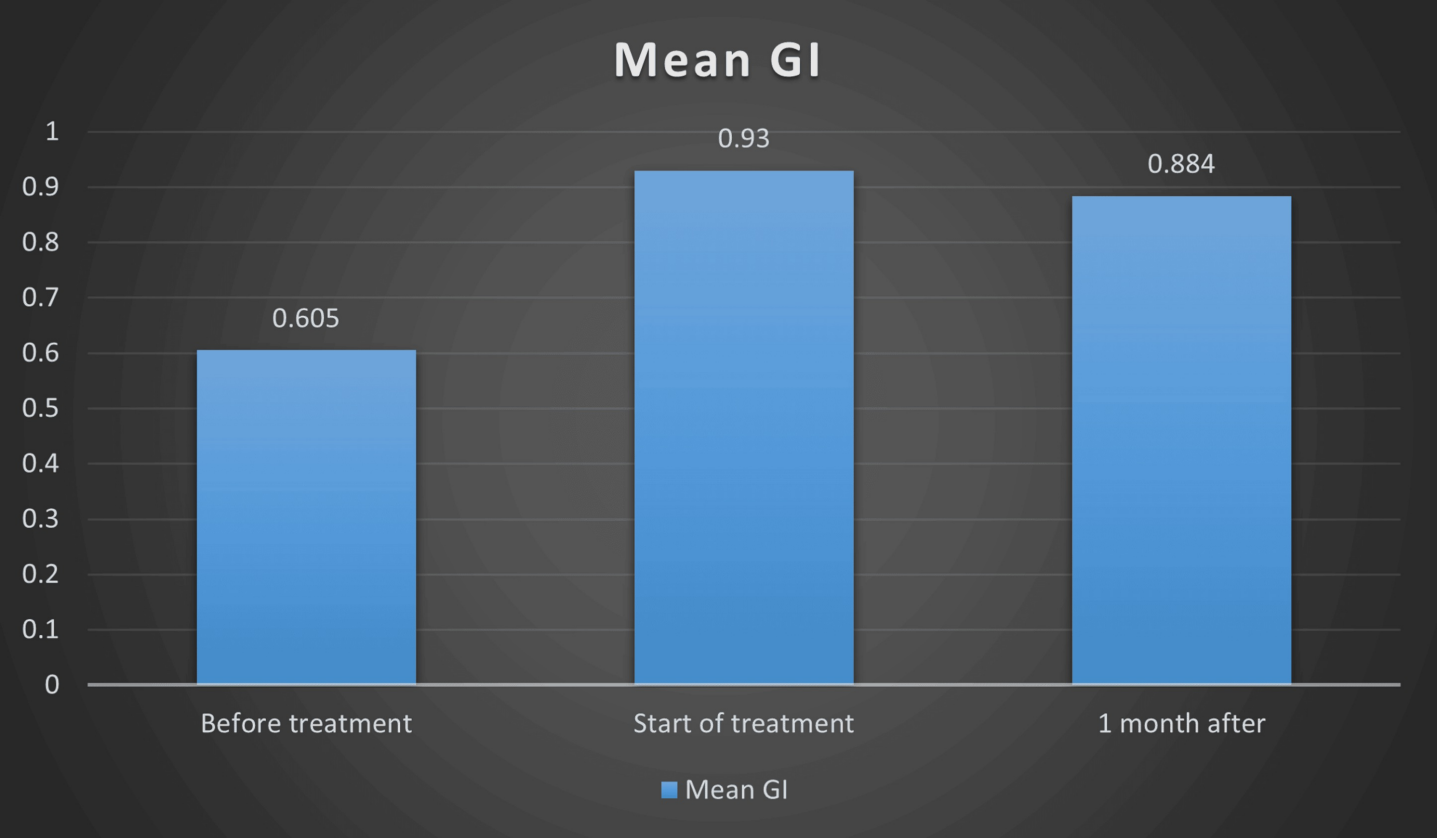
Mean gingival index (GI) scores at baseline, appliance placement, and one-month review

The intragroup comparison of GI scores showed a clear shift in gingival health after orthodontic appliance placement (Table 2, Figure 1). Before treatment, the mean GI was 0.605 ± 0.54, indicating generally mild inflammation. Once the fixed appliances were placed, the mean score increased to 0.93 ± 0.258, a change that was statistically significant (p = 0.001). This early rise is consistent with what is typically seen in practice, as brackets and wires create new areas for plaque to accumulate and may initially irritate the gingiva.

**Table 2:** Intragroup comparison of GI Friedman test: p < 0.05; ^*^Statistically significant (p < 0.05) GI: gingival index; NS: not significant

Intervals	Mean	SD	p-value	Wilcoxon signed-rank test
Baseline	0.605	0.54	0.003*	Baseline vs. start of treatment : p=0.001^*^
Start of treatment	0.93	0.258		Baseline vs. 1 month after p=0.006^*^
1 month after	0.884	0.625		Start of treatment vs. 1 month after : p=0.63

When the baseline scores were compared with those recorded one month after treatment began, the GI remained significantly higher (p = 0.006). However, there was no significant difference between the “start of treatment” and “one month” intervals (p = 0.63) (Table 2). This suggests that although the initial placement of appliances leads to a noticeable inflammatory response, the gingival condition begins to stabilize within the first month as patients adapt to their appliances and become more familiar with the required oral hygiene routine. The Friedman test supported these findings by showing an overall significant difference across all three time points (p < 0.05).

**Figure 2:**
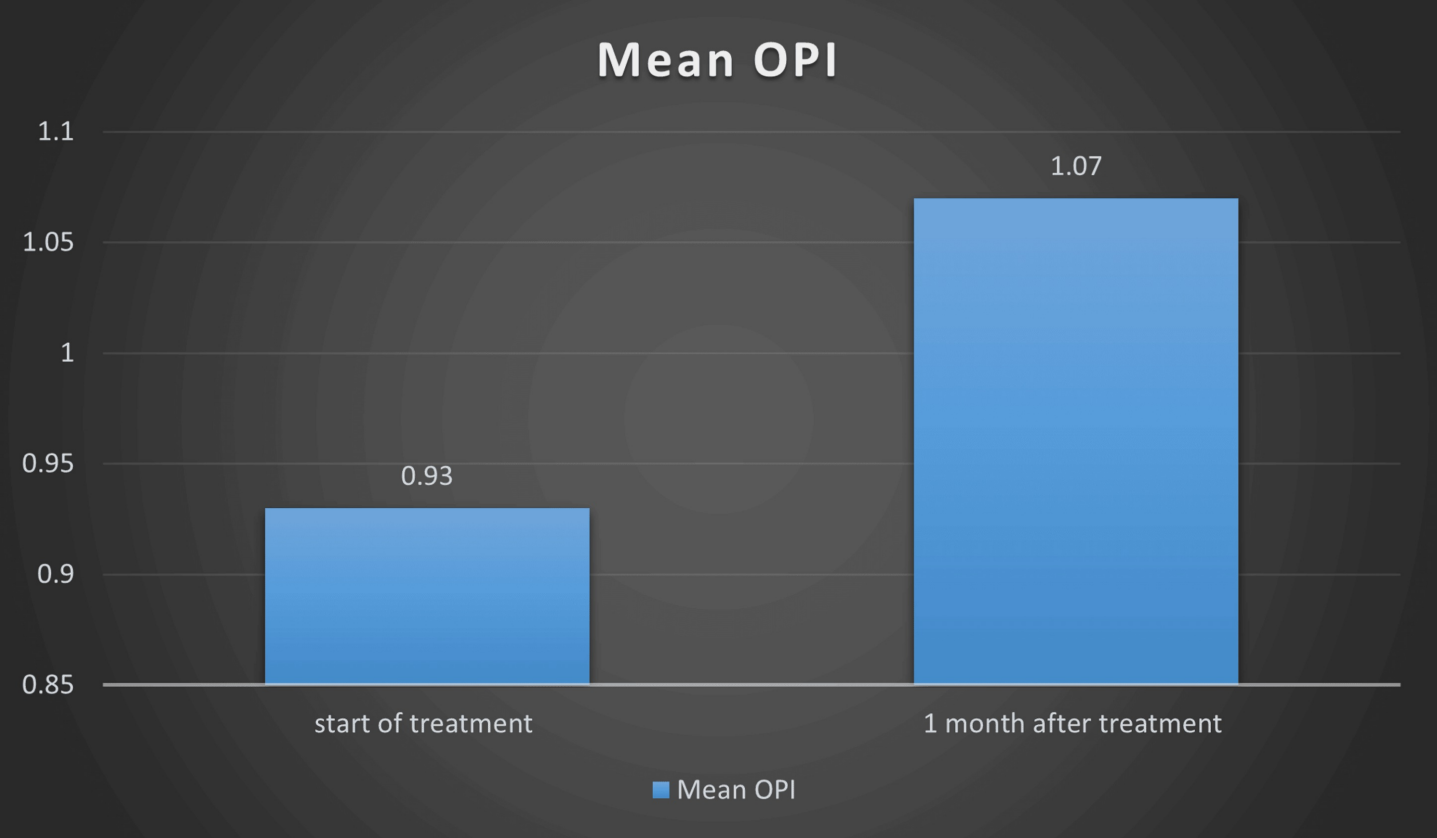
Mean orthodontic plaque index (OPI) scores at appliance placement and one-month review

The OPI showed a similar trend. The mean OPI increased from 0.93 ± 0.258 at the start of treatment to 1.07 ± 0.457 after one month, and this change was statistically significant (p < 0.05) (Table 3, Figure 2). This reflects the common challenge orthodontic patients face during the early treatment period: maintaining effective plaque control around brackets and archwires.

**Table 3:** Intragroup comparison of OPI Wilcoxon signed-rank test, p<0.05; ^*^Statistically significant

Intervals	Mean	SD	p-value
Start of treatment	0.93	0.258	0.02^*^
1 month after	1.07	0.457	

Taken together, the results indicate a predictable pattern during the early phase of fixed orthodontic therapy, an initial decline in gingival and oral hygiene status followed by gradual stabilization. These findings highlight the importance of early reinforcement of oral hygiene instructions, close follow-up appointments, and supportive preventive measures to help patients navigate this adjustment period successfully.

## Discussion

The present study examined early periodontal changes in patients undergoing fixed orthodontic treatment and found a significant increase in both the GI and OPI during the first month. This early deterioration in periodontal parameters is a well-recognized phenomenon and has been consistently reported across various populations.

The initial rise in GI observed in our cohort mirrors the findings of Marincak Vrankova et al. [14], who demonstrated that the placement of fixed appliances disrupts the oral environment, promoting early plaque retention and localized gingival inflammation. Their study highlighted that even in patients with relatively good baseline periodontal health, the introduction of brackets and wires alters the gingival microenvironment almost immediately. Similarly, Zhao et al. [15] showed that early appliance placement leads not only to clinical inflammation but also to a measurable shift in the oral microbiome, with increases in pathogenic bacterial species during the first few weeks. These microbial changes may help explain why inflammation rises quickly despite patient attempts to maintain oral hygiene.

Cerroni and colleagues [16] have also emphasized that fixed orthodontic appliances create new stagnation areas that are challenging for patients to clean effectively during the adaptation period. Their systematic review concluded that the early stage of treatment, particularly the first month, is the critical window during which gingival inflammation peaks before gradually stabilizing. This is in line with the pattern seen in our study, where the steepest increase occurred between baseline and appliance placement, with a subsequent plateau from appliance placement to the one-month follow-up.

A prospective study by Rakhshan and Rakhshan [17] also supports our findings, noting that the early phase of treatment is characterized by a predictable but temporary rise in plaque accumulation and gingival bleeding scores. They attributed this to mechanical irritation caused by archwires and bracket wings, combined with a lapse in patients' oral hygiene efficiency as they adapt to new brushing paths. Dixit et al. [18] reported a similar pattern, observing that GI values generally increase during the first 4-6 weeks and tend to stabilize thereafter, especially in individuals who receive repeated oral hygiene reinforcement, a factor that may also account for the stabilization observed in our one-month follow-up.

In addition to individual clinical studies, higher-level evidence supports this concept. Di Spirito et al. [19], in a recent umbrella review, observed that fixed appliances have a significantly greater short-term impact on gingival health compared with removable appliances or aligners. This reinforces the idea that early mechanical and microbial disturbances associated with fixed appliances are largely unavoidable, even in motivated patients.

The stabilization in GI values after the initial peak, as seen in our results, suggests that the earliest phase of treatment is when patients are most vulnerable to periodontal changes. This underlines the importance of early intervention strategies such as personalized oral hygiene instruction, motivational reinforcement, and the use of adjunctive tools like interdental brushes, orthodontic toothbrushes, and antimicrobial mouth rinses. Studies consistently show that such strategies can blunt the peak of early gingival inflammation and prevent long-term periodontal complications.

What makes our study noteworthy is its focus on the very first month of treatment, as our findings show that clinically meaningful changes can be detected much earlier. This emphasizes the importance of early follow-up appointments, patient education, and the immediate use of hygiene aids to protect gingival and periodontal health from the outset of treatment.

Clinical implications

Overall, the strong concordance between our findings and previously published literature [14-19] indicates that early periodontal changes during fixed orthodontic therapy are consistent, predictable, and largely manageable with proper preventive care. For clinicians, the key message is clear: gingival health can decline rapidly after appliance placement, but this decline is both predictable and preventable. By emphasizing preventive measures from day one, encouraging interdental cleaning, and scheduling early reviews, orthodontists can help patients maintain healthier gingiva and improve long-term treatment outcomes.

Limitations and future research

This study has some limitations, encompassing a relatively modest sample size, a single-center setting, and a short follow-up period. While we demonstrated clear early changes, longer-term and larger studies are needed to determine whether these changes stabilize, worsen, or improve with time. Future research should also compare different appliance systems, including ceramic brackets, self-ligating brackets, and aligners, and evaluate preventive strategies such as powered toothbrushes, probiotics, and newer antimicrobial approaches.

## Conclusions

Our findings show that gingival health can decline quickly once fixed orthodontic appliances are placed. At baseline and at appliance placement, conditions were stable, but within just a month, both gingival inflammation and plaque accumulation had increased significantly. This underscores how brackets, wires, and ligatures act as strong plaque-retentive factors, making everyday cleaning more difficult for patients.

For orthodontic care providers, the lesson is clear: prevention needs to begin from day one. Reinforcing oral hygiene, encouraging the use of interdental brushes or irrigators, and monitoring patients closely at each visit can go a long way in protecting gingival health during treatment.

## References

[1] Zachrisson BU (1976). Cause and prevention of injuries to teeth and supporting structures during orthodontic treatment. Am J Orthod.

[2] Gorelick L, Geiger AM, Gwinnett AJ (1982). Incidence of white spot formation after bonding and banding. Am J Orthod.

[3] van Gastel J, Quirynen M, Teughels W, Coucke W, Carels C (2008). Longitudinal changes in microbiology and clinical periodontal variables after placement of fixed orthodontic appliances. J Periodontol.

[4] Lucchese A, Bondemark L, Marcolina M, Manuelli M (2018). Changes in oral microbiota due to orthodontic appliances: a systematic review. J Oral Microbiol.

[5] Guo R, Lin Y, Zheng Y, Li W (2017). The microbial changes in subgingival plaques of orthodontic patients: a systematic review and meta-analysis of clinical trials. BMC Oral Health.

[6] Löe H, Silness J (1963). Periodontal disease in pregnancy. I. Prevalence and severity. Acta Odontol Scand.

[7] Sukontapatipark W, el-Agroudi MA, Selliseth NJ, Thunold K, Selvig KA (2001). Bacterial colonization associated with fixed orthodontic appliances. A scanning electron microscopy study. Eur J Orthod.

[8] Lara-Carrillo E, Montiel-Bastida NM, Sánchez-Pérez L, Alanís-Tavira J (2010). Effect of orthodontic treatment on saliva, plaque and the levels of Streptococcus mutans and Lactobacillus. Med Oral Patol Oral Cir Bucal.

[9] Arab S, Nouhzadeh Malekshah S, Abouei Mehrizi E, Ebrahimi Khanghah A, Naseh R, Imani MM (2016). Effects of fixed orthodontic treatment on salivary flow, pH and microbial count. J Dent (Tehran).

[10] Chapple IL, Mealey BL, Van Dyke TE (2018). Periodontal health and gingival diseases and conditions on an intact and a reduced periodontium: Consensus report of workgroup 1 of the 2017 World Workshop on the Classification of Periodontal and Peri-Implant Diseases and Conditions. J Periodontol.

[11] Faul F, Erdfelder E, Lang AG, Buchner A (2007). G*Power 3: a flexible statistical power analysis program for the social, behavioral, and biomedical sciences. Behav Res Methods.

[12] Yadav J, Shinh AS, Natt AS, Maheshwari K, Aulakh S (2023). Oral hygiene status: The critical parameter in orthodontic patient. J Clin Adv Dent.

[13] Heintze SD, Jost-Brinkmann PG, Finke C, Miethke RR (1999). Oral Health for the Orthodontic Patient, 1st ed. Oral Health for the Orthodontic Patient. 1st ed. Hanover Park, Ill: Quintessence Publishing Co.

[14] Marincak Vrankova Z, Rousi M, Cvanova M (2022). Effect of fixed orthodontic appliances on gingival status and oral microbiota: a pilot study. BMC Oral Health.

[15] Zhao M, Yu C, Su C (2024). Dynamic effects of fixed orthodontic treatment on oral health and oral microbiota: a prospective study. BMC Oral Health.

[16] Cerroni S, Pasquantonio G, Condò R, Cerroni L (2018). Orthodontic fixed appliance and periodontal status: an updated systematic review. Open Dent J.

[17] Rakhshan H, Rakhshan V Effects of the initial stage of active fixed orthodontic treatment and sex on dental plaque accumulation: A preliminary prospective cohort study. Saudi J Dent Res.

[18] Dixit A, Paul S, Lakhani S, Badiyani BK, Arora NN, Arya R, Kumar A (2023). A study to assess and evaluate the gingival response during and after the fixed orthodontics treatment experienced by adult patients. J Pharm Bioallied Sci.

[19] Di Spirito F, D'Ambrosio F, Cannatà D, D'Antò V, Giordano F, Martina S (2023). Impact of clear aligners versus fixed appliances on periodontal status of patients undergoing orthodontic treatment: a systematic review of systematic reviews. Healthcare (Basel).

